# Decreased Expression of IL‐35 and Its Receptor Contributes to Impaired Megakaryopoiesis in the Pathogenesis of Immune Thrombocytopenia

**DOI:** 10.1002/advs.202305798

**Published:** 2024-01-15

**Authors:** Xuan Cai, Ruo‐Yun Gui, Jin Wu, Chen‐Cong Wang, Xiao‐Lu Zhu, Hai‐Xia Fu, Xiao‐Hui Zhang

**Affiliations:** ^1^ Peking University People's Hospital Beijing 100044 China; ^2^ Peking University Institute of Hematology Beijing 100044 China; ^3^ National Clinical Research Center for Hematologic Disease Beijing 100044 China; ^4^ Beijing Key Laboratory of Hematopoietic Stem Cell Transplantation Beijing 100044 China

**Keywords:** iguratimod, IL‐35, iTr35, megakaryopoiesis, mesenchymal stem cells

## Abstract

Recent findings have shown that the level of interleukin‐35 (IL‐35) is abnormal in several autoimmune diseases. Nonetheless, whether IL‐35 participates in the pathogenesis of immune thrombocytopenia (ITP) remains unclear. The current study investigates whether IL‐35 modulates megakaryopoiesis. The results show that IL‐35 receptors are progressively expressed on bone marrow megakaryocytes during the in vitro differentiation of CD34+ progenitors. IL‐35 increases the number of megakaryocyte colony‐forming units through the Akt pathway. The level of bone marrow IL‐35 is reduced in ITP patients, and the decreased level of IL‐35 may inhibit megakaryopoiesis. Then, the potential causes of decreased IL‐35 in ITP patients are explored. The primary type of cell that secretes IL‐35, known as IL‐35‐producing regulatory T cells (iTr35), is reduced in ITP patients. Bone marrow mesenchymal stem cells (MSCs) from ITP patients exhibit an impaired capability of inducing iTr35 due to enhanced apoptosis, which may contribute to the reduced level of bone marrow IL‐35 in ITP patients. Iguratimod promotes megakaryocyte development and differentiation by elevating the expression of IL‐35 receptors on megakaryocytes. Iguratimod improves response rates and reduces bleeding symptoms in corticosteroid‐resistant ITP patients.

## Graphical abstract

Proposed model of decreased IL‐35 in ITP bone marrow and its potential mechanism in the pathogenesis of ITP. ITP‐MSCs in bone marrow exhibit an impaired capability of inducing iTr35, which may contribute to the reduced level of bone marrow IL‐35 in ITP patients. IL‐35 receptors are progressively induced on megakaryocytes, and IL‐35 promotes megakaryopoiesis through the Akt pathway. Iguratimod promotes megakaryopoiesis by elevating the expression of IL‐35 receptors on megakaryocytes.

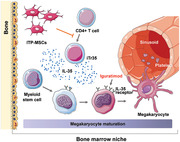

## 1 Introduction

Previous findings showed that the levels of plasma and bone marrow interleukin‐35 (IL‐35) in immune thrombocytopenia (ITP) patients declined markedly in comparison with those in healthy controls.^[^ ^1^, ^2^, ^3^ ^]^ IL‐35 is an inhibitory cytokine of the IL‐12 family, which comprises an α chain (p35 subunit) and a β chain (Epstein‐Barr virus‐induced gene 3).^[^ ^4^, ^5^ ^]^ IL‐35 is produced by several immune cells, such as regulatory T cells (Tregs) and regulatory B cells (Bregs).^[^ ^1^, ^6^, ^7^, ^8^, ^9^, ^10^ ^]^ A new type of Treg, known as IL‐35‐producing Treg (iTr35) (IL‐35+CD4+IL‐10‐TGFβ‐Foxp3‐), has been revealed as the major source of IL‐35.^[^ ^4^, ^11^ ^]^ IL‐35 functions through the IL‐35 receptor, which is composed of a heterodimer of IL‐12Rβ2 and gp130 or homodimers of each chain.^[^ ^12^, ^13^ ^]^ IL‐35 potently limits inflammatory responses predominantly through suppressing T‐cell proliferation and differentiation and inducing naive T cells into Treg cells.^[^ ^4^, ^12^, ^14^, ^15^ ^]^ IL‐35 also prevents the secretion of inflammatory cytokines from T cells, such as IL‐12, interferon‐γ and IL‐17.^[^ ^2^, ^16^, ^17^, ^18^ ^]^ In addition, IL‐35 suppresses the expansion of B cells and induces IL‐10‐producing B cells.^[^ ^19^ ^]^

Several studies have investigated the role of IL‐35 in ITP. The level of plasma IL‐35 in ITP patients was positively correlated with the percentage of peripheral Tregs and the number of platelet counts, and the IL‐35 level showed a negative correlation with Th1 cells in ITP patients.^[^ ^3^ ^]^ In addition, IL‐35 reduced the percentage of CD4+ T cells but induced the proliferation of Tregs in ITP patients.^[^ ^1^, ^20^ ^]^ Peripheral IL‐35 in ITP patients was upregulated after intravenous immunoglobulin and high‐dose dexamethasone treatment.^[^ ^21^, ^22^ ^]^ Moreover, the concentration of plasma IL‐35 in ITP patients achieving complete response returned to normal but was not corrected in those who failed to achieve response.^[^ ^21^, ^23^ ^]^ However, the underlying mechanism of IL‐35 in ITP remains unclear.

As a polyphenolic flavonoid extracted from fenugreek seeds, iguratimod is a safe and effective disease‐modifying antirheumatic drug that has been widely applied in rheumatoid arthritis (RA) patients in China and Japan.^[^ ^24^, ^25^, ^26^, ^27^, ^28^ ^]^ Iguratimod has also been shown to suppress proinflammatory cytokine production and inhibit the signaling of several interleukins.^[^ ^29^, ^30^, ^31^, ^32^ ^]^ In addition, iguratimod could dampen T‐cell proliferation and suppress B‐cell terminal differentiation, as well as immunoglobulin production from plasma cells.^[^ ^31^, ^33^ ^]^ Furthermore, iguratimod upregulates the Treg response and increases the levels of Treg‐associated transcription factors and cytokines.^[^ ^28^, ^34^ ^]^ ITP is an autoimmune disease in which T and B cells play critical roles in the underlying pathogenesis, implying that iguratimod may have the potential for the treatment of ITP. Nonetheless, no previous study investigating the clinical outcomes of iguratimod in ITP patients has been published. As mentioned above, the level of IL‐35 decreased in ITP patients, which might arise from abnormal IL‐35‐producing Tregs. Therefore, in this study, we investigated whether iguratimod could elevate the level of iTr35 cells in ITP.

In the current research, we sought to assess the level of bone marrow IL‐35 and its physiopathologic mechanism in ITP. Furthermore, we investigated whether iguratimod could rectify the decreased level of IL‐35 in ITP bone marrow. In addition, a retrospective study was performed to evaluate the clinical outcomes of iguratimod in corticosteroid‐resistant ITP patients.

## 2 Results

### 2.1 Reduced IL‐35 in ITP Patients

**Figure 1:**
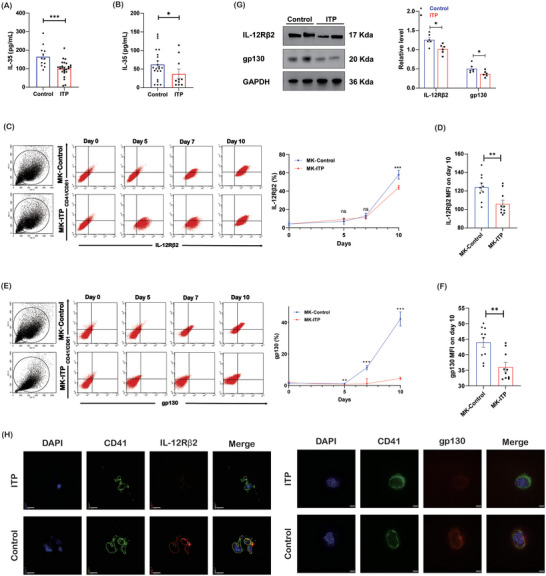
Reduced IL‐35 in patients with ITP. A) The level of plasma IL‐35 by ELISA (n = 12 in the control group, n = 26 in the ITP group). B) The concentration of IL‐35 in the bone marrow of ITP patients and healthy controls by ELISA (n = 22 in the control group, n = 10 in the ITP group). C) Expression of IL‐35 receptor subunit IL‐12Rβ2 in CD41/CD61+ cells by flow cytometry (n = 10). D) The mean fluorescence intensity of IL‐12Rβ2 on day 10 by flow cytometry (n = 10). E) Expression of IL‐35 receptor subunit gp130 in CD41/CD61+ cells by flow cytometry (n = 10). F) The mean fluorescence intensity of gp130 on day 10 by flow cytometry (n = 10). G) The protein levels of IL‐35 receptor subunits IL‐12Rβ2 and gp130 in CD41/CD61+ cells (n = 6). H) CD41 (green), IL‐12Rβ2/ gp130 (red), and DAPI (blue) staining of CD41/CD61+ cells (600×; bar: 20 µm). Data information: Data are presented as mean ± SEM. ^*^ *p* < 0.05, ^**^ *p* < 0.01, ^***^ *p* < 0.001; ns, no significant difference, using Student‘s t‐test (A, B, D, F) or ANOVA (C, E).

In comparison with that in healthy controls, the plasma level of IL‐35 was lower in ITP patients (165.30 ± 16.49 vs 101.30 ± 8.31 pg ml^−1^, *p* < 0.001, **Figure** **1**A). Bone marrow IL‐35 was also lower in ITP (58.56 ± 7.38 vs 37.52 ± 5.33 pg ml^−1^, *p* = 0.02, Figure 1B), which suggested that IL‐35 might play a part in ITP. We further examined the effect of IL‐35 on megakaryocyte differentiation. Bone marrow CD34+ cells from ITP patients and healthy controls were differentiated into megakaryocytes with thrombopoietin (TPO) alone. Flow cytometry analysis of CD41/CD61+ cells indicated that IL‐35 receptors (IL‐12Rβ2 and gp130) were progressively induced in both groups during megakaryopoiesis. The expression of the IL‐35 receptor subunit IL‐12Rβ2 was comparable between ITP patients and healthy controls in the first 7 days (Figure 1C). However, the level of IL‐12Rβ2 was lower in ITP patients on day 10 (Figure 1C,D). The expression of gp130 was also lower in ITP patients on day 10 (Figure 1E,F). The lower levels of IL‐12Rβ2 and gp130 in ITP were confirmed by western blot analysis (Figure 1G). Immunofluorescence presented the expression of IL‐35 receptor subunits on day 10 (Figure 1H). These results revealed that the expression of IL‐35 receptors was reduced in megakaryocytes from ITP patients compared with that from healthy controls.

### 2.2 IL‐35 Stimulates Megakaryopoiesis In Vitro

**Figure 2:**
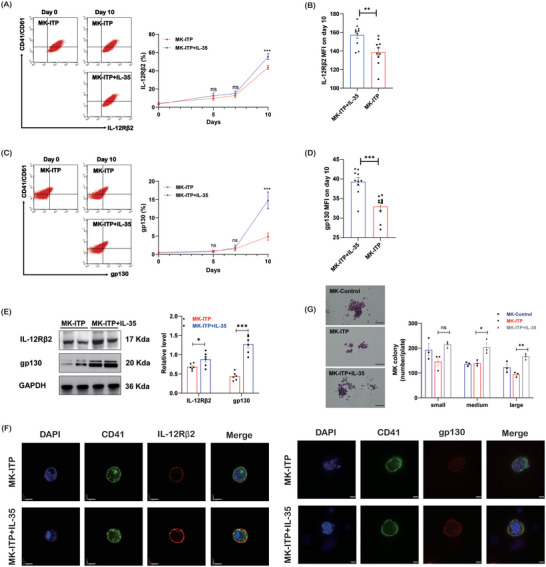
IL‐35 stimulates megakaryopoiesis in vitro. A) Expression of IL‐35 receptor subunit IL‐12Rβ2 in CD41/CD61+ cells from ITP patients by flow cytometry (n = 10). B) The mean fluorescence intensity of IL‐12Rβ2 on day 10 by flow cytometry (n = 10). C) Expression of IL‐35 receptor subunit gp130 in CD41/CD61+ cells from ITP patients by flow cytometry (n = 10). D) The mean fluorescence intensity of gp130 on day 10 by flow cytometry (n = 10). E) The protein levels of IL‐35 receptor subunits IL‐12Rβ2 and gp130 in CD41/CD61+ cells (n = 6). F) CD41 (green), IL‐12Rβ2/ gp130 (red), and DAPI (blue) staining of CD41/CD61+ cells (600×; bar: 20 µm). G) (Left) Representative images of megakaryocyte colonies (100×; bar: 100 µm). (Right) The number of megakaryocyte colonies (n = 3). Data information: Data are presented as mean ± SEM. ^*^ *p* < 0.05, ^**^ *p* < 0.01, ^***^ *p* < 0.001; ns, no significant difference, using ANOVA (A, C, E, F) or Student‘s t‐test (B, D).

We then evaluated the effect of IL‐35 on the differentiation of megakaryocytes. CD34+ cells from ITP bone marrow were differentiated into megakaryocytes with TPO and IL‐35. IL‐35 increased the expression of IL‐12Rβ2 (**Figure** **2**A,B) and gp130 (Figure 2C,D) on megakaryocytes on day 10. The higher levels of IL‐12Rβ2 and gp130 in the IL‐35 group were confirmed by western blot analysis (Figure 2E). Immunofluorescence showed similar results for IL‐35 receptors on day 10 (Figure 2F). We then measured the effect of IL‐35 on the formation of colony‐forming units. IL‐35 did not affect the number of small colonies but increased the number of medium‐sized and large colonies (Figure 2G). These data showed that the expression of IL‐35 receptors on megakaryocytes from ITP patients was decreased, which was reversed by IL‐35. IL‐35 promoted megakaryopoiesis and increased the number of megakaryocyte colonies in vitro.

**Figure 3:**
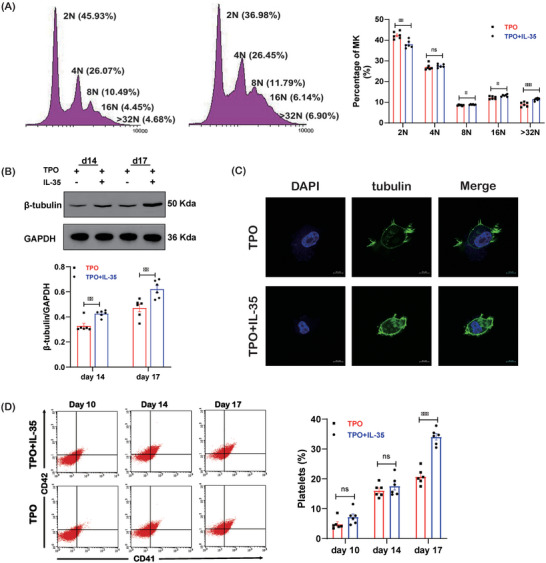
IL‐35 promotes the in vitro proliferation of megakaryocytes. A) Megakaryocyte ploidy was analyzed by flow cytometry on day 17 (n = 6). B) The protein levels of β‐tubulin in megakaryocytes (n = 6). C) β‐tubulin (green) and DAPI (blue) staining of megakaryocytes (1000×; bar: 10 µm). D) Platelet production from megakaryocytes was analyzed by flow cytometry (n = 6). Data information: Data are presented as mean ± SEM. ^*^ *p* < 0.05, ^**^ *p* < 0.01, and ^***^ *p* < 0.001; ns, no significant difference, using Student's t‐test.

Megakaryocyte ploidy correlates with megakaryocyte maturation. IL‐35 treatment caused higher ploidy (**Figure** **3**A). There were fewer 2N megakaryocytes and more 8N+ megakaryocytes in the IL‐35 group than in the TPO alone group. The percentage of 4N megakaryocytes was similar between the two groups. Increased expression of β‐tubulin protein was also observed in megakaryocytes treated with IL‐35 (Figure 3B,C). IL‐35 stimulation induced a significant increase in platelet release from megakaryocytes on day 17 (Figure 3D). Platelets from the TPO+IL‐35 group were similar in size and identical in discoid morphology to platelets from the TPO group (Figure S1A, Supporting Information). Platelet aggregation studies showed that platelets in the TPO group and TPO+IL‐35 group exhibited similar aggregation responses (Figure S1B, Supporting Information).

### 2.3 IL‐35 Promotes the Differentiation of Megakaryocytes Through the Akt Pathway

**Figure 4:**
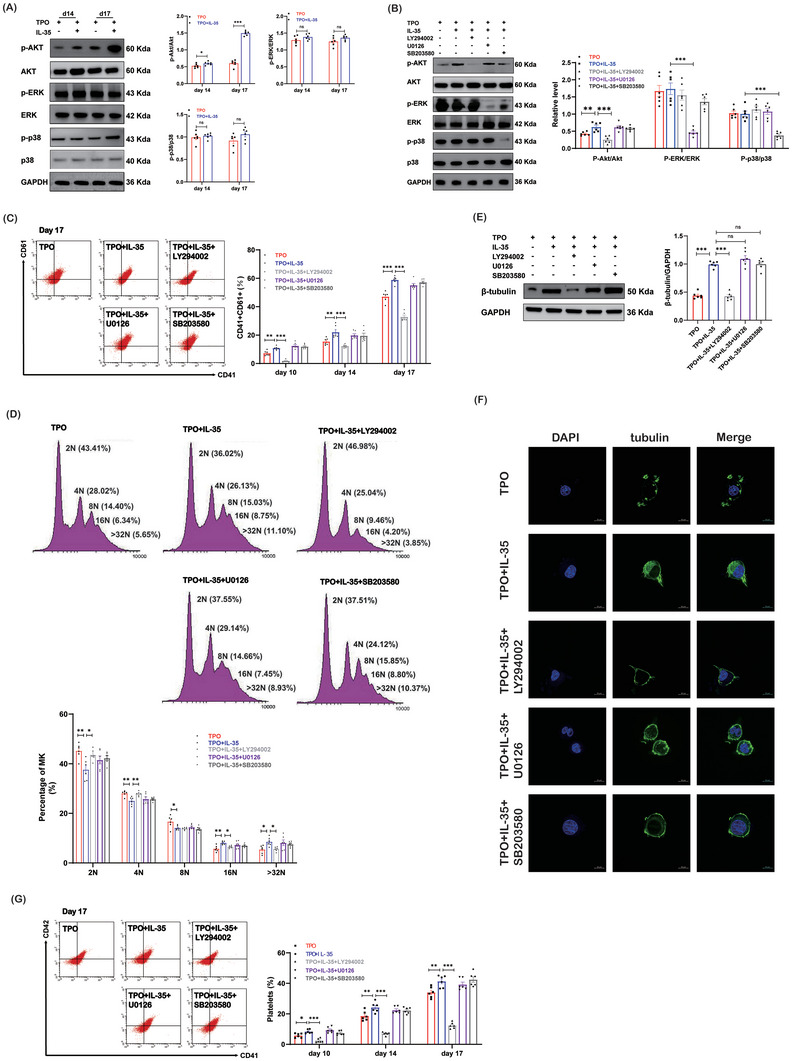
IL‐35 promotes megakaryopoiesis via Akt activity. A) The protein levels of Akt, ERK, and p38 phosphorylation in megakaryocytes (n = 6). B) The protein levels of Akt, ERK and p38 phosphorylation in megakaryocytes pretreated with specific inhibitors (n = 6). C) Cells pretreated with specific inhibitors are labeled with anti‐CD41 and anti‐CD61 antibodies, and analyzed by flow cytometry on day 10, 14, and 17 culture (n = 6). D) Megakaryocyte ploidy was analyzed by flow cytometry with specific inhibitors on day 17 (n = 6). E) The protein levels of β‐tubulin in megakaryocytes (n = 6). F) β‐tubulin (green) and DAPI (blue) staining of megakaryocytes (1000×; bar: 10 µm). G) Platelet production from megakaryocytes pretreated with specific inhibitors on day 10, 14, and 17 culture (n = 6). Data information: Data are presented as mean ± SEM. ^*^ *p* < 0.05, ^**^ *p* < 0.01, and ^***^ *p* < 0.001; ns, no significant difference, using ANOVA.

We then focused on the signaling pathways through which IL‐35 regulated the differentiation of megakaryocytes and platelet production. As shown, a higher level of Akt phosphorylation was observed after IL‐35 stimulation (**Figure** **4**A). The levels of phosphorylated ERK and p38 were similar between the TPO group and the TPO plus IL‐35 group (Figure 4A). Akt phosphorylation was inhibited by pretreatment with the inhibitor LY294002. ERK phosphorylation was inhibited by pretreatment with the inhibitor U0126. The p38 phosphorylation was inhibited by pretreatment with the inhibitor SB203580 (Figure 4B). Pretreatment with LY294002 blocked the differentiation of megakaryocytes induced by IL‐35 (Figure 4C). There were fewer 2N and 4N megakaryocytes in the IL‐35 group than in the LY294002 group (Figure 4D). There were more 16N+ megakaryocytes in the IL‐35 group than in the LY294002 group. The percentage of 8N megakaryocytes was similar between the IL‐35 group and the LY294002 group. Pretreatment with U0126 or SB203580 did not affect megakaryopoiesis (Figure 4C,D). LY294002 decreased the expression of β‐tubulin in megakaryocytes stimulated by IL‐35, which was not observed in the U0126 or SB203580 pretreatment group (Figure 4E,F). Flow cytometry analysis revealed that platelet production decreased in the LY294002 pretreatment group (Figure 4G). These results demonstrated that IL‐35 could enhance the proliferation and differentiation of megakaryocytes through the Akt pathway.

### 2.4 ITP‐MSCs Exhibit an Impaired Capability of Inducing iTr35

**Figure 5:**
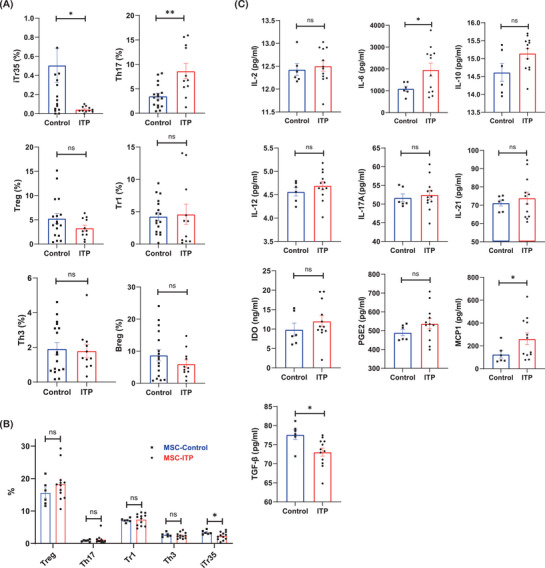
ITP‐MSCs show impaired immunosuppressive properties. A) Flow cytometry analysis of bone marrow T cell subsets in ITP patients and healthy controls (n = 17 in the control group, n = 11 in the ITP group). B) Flow cytometry analysis of T cell subsets when cocultured with MSCs from ITP patients and healthy controls (n = 6 in the control group, n = 12 in the ITP group). C) The concentration of cytokines in the coculture supernatant of T cells and MSCs from ITP patients and healthy controls by ELISA (n = 6 in the control group, n = 12 in the ITP group). Data information: Data are presented as mean ± SEM. ^*^ *p* < 0.05, ^**^ *p* < 0.01, and ^***^ *p* < 0.001; ns, no significant difference, using Student's t‐test.

It has been shown that IL‐35 in plasma and bone marrow is decreased in ITP patients. We then investigated the potential causes of the reduced IL‐35. The iTr35 cells have been shown to be the primary type of cells that secrete IL‐35.^[^ ^4^ ^]^ CD8+ Tregs, Bregs, and tolerogenic dendritic cells are also cellular sources of IL‐35.^[^ ^1^, ^6^, ^10^, ^11^ ^]^ We then analyzed the composition of bone marrow lymphocytes in ITP patients. Flow cytometry analysis showed that the percentage of bone marrow iTr35 cells was significantly lower in ITP patients, and the percentage of Th17 cells was higher in ITP patients than in healthy controls (**Figure** **5**A). The percentages of Tr1, Treg, Th3, and Breg cells were comparable between the two groups. Thus, we postulated that the decreased bone marrow iTr35 cells might contribute to the reduced level of IL‐35 in ITP bone marrow. The immunomodulatory properties of MSCs on a variety of immune cells have been widely studied.^[^ ^35^, ^36^ ^]^ Thus, we cocultured CD4+ T cells with MSCs from healthy controls and ITP patients. The percentage of iTr35 cells was lower in the ITP group than in the control group (Figure 5B). There was no difference in other T‐cell subsets (Figure 5B). These results showed that ITP‐MSCs exhibited an impaired capability of inducing iTr35. MSCs mediate the differentiation of T cells mainly through soluble cytokines. We determined the concentration of cytokines in coculture supernatants. The level of TGF‐β was lower in the ITP group, and the levels of MCP1 and IL‐6 were higher in the ITP group than in the control group (Figure 5C).

### 2.5 Identification of Aberrantly Expressed Transcripts in ITP‐MSCs

**Figure 6:**
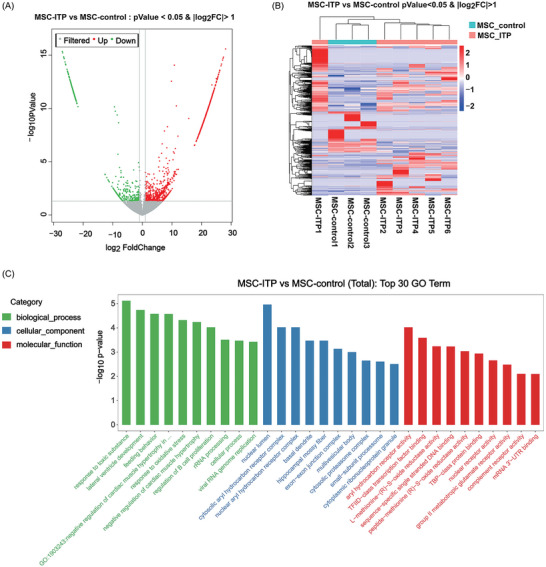
The relative expression levels of lncRNAs in ITP‐MSCs. A) Volcano plot of differentially expressed lncRNAs in MSCs from ITP patients and healthy controls. Red color represents upregulated lncRNAs and the green color represents downregulated lncRNAs in ITP‐MSCs (the cutoff values of *p* < 0.05 and ∣logFC∣>2). B) Heatmap plot of the differently expressed lncRNAs. Color keys from blue to red indicate relative expression levels from low to high. C) GO pathways of differentially expressed lncRNAs. D) KEGG analysis of differentially expressed lncRNAs. The vertical axis on the left represents the name of the top pathways, and the horizontal axis at the bottom represents the degree of clustering of samples. The P‐value represents the degree of clustering. E) The ITP‐associated lncRNA‐TF‐mRNA regulatory network of bone marrow MSCs. The red nodes represent lncRNAs, the green triangles represent mRNA, and the blue squares represent TF, and size increases with the degree. F) Relative expression of genes analyzed by PCR (n = 6). G) The protein level of FAM107A in MSCs (n = 6). H) The mRNA level of FAM107A in MSCs (n = 6). I) The protein level of FAM107A in MSCs (n = 6). J) CCK‐8 assay of MSCs (n = 6). K) Cell apoptosis was examined by flow cytometry (n = 6). L) The protein level of apoptosis markers (n = 6). M) The proportion of iTr35 in CD4+ cells cocultured with MSCs detected by flow cytometry (n = 6). Data information: Data are presented as mean ± SEM. ^*^ *p* < 0.05, ^**^ *p* < 0.01, and ^***^ *p* < 0.001; ns, no significant difference, using Student's t‐test (F, G, H, I) or ANOVA (J, K, L, M).

Long noncoding RNAs (lncRNAs) may facilitate the progression of various autoimmune disorders by regulating vital biological processes. To gain deeper insight, we characterized the profile of lncRNAs in bone marrow MSCs from ITP patients and healthy controls using RNA sequencing. A total of 1324 and 507 lncRNAs were significantly upregulated or downregulated, respectively, in ITP‐MSCs (**Figure** **6**A,B). Gene Ontology (GO) analysis (Figure 6C) indicated that the biological processes associated with the differential lncRNAs were response to toxic substances, response to oxidative stress, and regulation of B‐cell proliferation. Regarding the cellular components, the differential lncRNAs were mainly related to the nuclear lumen, cytosolic aryl hydrocarbon receptor complex, and nuclear aryl hydrocarbon receptor complex. Moreover, the molecular functions mainly associated with the differential lncRNAs were aryl hydrocarbon receptor activity, TFIID‐class transcription factor binding, and L‐methionine‐(R)‐S‐oxide reductase activity. Kyoto Encyclopedia of Genes and Genomes (KEGG) analysis demonstrated the important pathways, including cell adhesion molecules (Figure 6D). To further enrich the regulatory mechanism, we constructed a lncRNA‐transcriptional factors (TFs)‐mRNA network by overlapping mRNAs co‐expressed with the key lncRNAs and target genes of TFs. Based on the coexpression network, 2 lncRNAs coexpressed with mRNAs as well as overlapping with the target genes of a specific TF were included in the network (Figure 6E). We found that ZSCAN31, FAM107A, U2AF1L5, DUSP26, MAGEA4, TACSTD2, MYL2, MYH6, MYH7, and SLITRK5 were differentially expressed in ITP‐MSCs. To validate this finding, the expression of the 10 genes was further confirmed by PCR (Figure 6F). The expression of TACSTD2, MYL2, MYH6, MYH7, and SLITRK5 was not significantly different between the ITP‐MSCs and control groups. There were statistical differences in the expression of ZSCAN31, U2AF1L5, DUSP26, MAGEA4, and FAM107A, which was consistent with the sequencing results. The expression of FAM107A increased in ITP‐MSCs compared with that in the controls, while the expression of ZSCAN31, U2AF1L5, DUSP26, and MAGEA4 decreased in ITP‐MSCs. Thus, we ultimately narrowed our analysis to FAM107A and deduced that FAM107A might be correlated with the pathogenesis of impaired MSCs. Similarly, the protein expression of FAM107A increased in ITP‐MSCs (Figure 6G). Our previous study demonstrated that ITP‐MSCs did not exhibit conventional proliferative abilities and thus exhibited defects in immunoregulation. Inhibiting apoptosis could restore the immunosuppressive capacity of ITP‐MSCs.^[^ ^37^ ^]^ The FAM107A gene has been revealed to induce cell apoptosis.^[^ ^38^ ^]^ Accordingly, we hypothesized that FAM107A might impair the immunosuppressive capacity of ITP‐MSCs by inducing MSCs apoptosis. To further verify this hypothesis, the FAM107A overexpression lentivirus was transfected into control MSCs. FAM107A expression was upregulated at the mRNA and protein levels in control‐MSCs subjected to FAM107A overexpression lentivirus (Figure 6H,I). The CCK‐8 assay showed that the cell proliferation ability of the FAM107A group was significantly decreased compared to that of the control group, indicating that the overexpression of FAM107A inhibited the proliferation and growth of MSCs (Figure 6J). Flow cytometry revealed that the proportions of apoptotic cells in the FAM107A group and ITP‐MSCs were higher than those in the control group (Figure 6K). Overexpression of FAM107A resulted in a decreased Bcl‐2/Bax ratio and increased cleaved caspase‐9 and caspase‐3 in control‐MSCs, similar to ITP‐MSCs (Figure 6L). Then, we cocultured CD4+ T cells with MSCs from the FAM107A group. The percentage of iTr35 cells was lower in the FAM107A group (Figure 6M). The results indicated that FAM107A impaired the immunosuppressive capacity of MSCs by inducing cell apoptosis.

### 2.6 Iguratimod Stimulates the Expression of IL‐35 Receptors on Megakaryocytes

**Figure 7:**
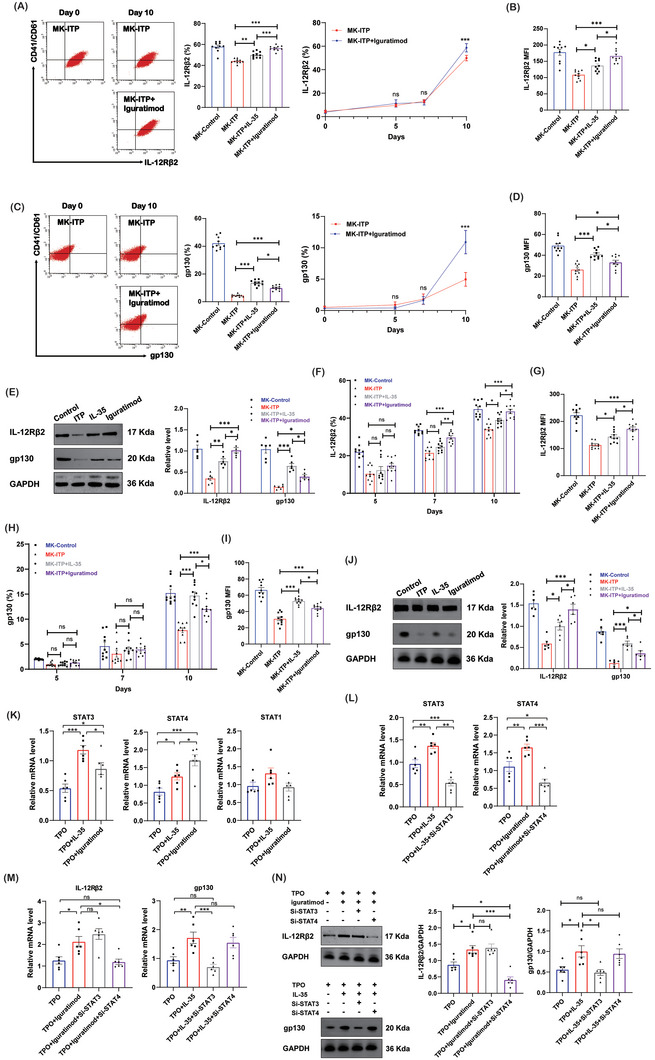
Iguratimod stimulates the expression of IL‐35 receptors on megakaryocytes. A) Expression of IL‐35 receptor subunit IL‐12Rβ2 in CD41/CD61+ cells from ITP patients by flow cytometry (n = 10). B) The mean fluorescence intensity of IL‐12Rβ2 on day 10 by flow cytometry (n = 10). C) Expression of IL‐35 receptor subunit gp130 in CD41/CD61+ cells from ITP patients by flow cytometry (n = 10). D) The mean fluorescence intensity of gp130 on day 10 by flow cytometry (n = 10). E) The protein levels of IL‐35 receptor subunits IL‐12Rβ2 and gp130 in CD41/CD61+ cells (n = 6). F) Expression of IL‐35 receptor subunit IL‐12Rβ2 in CD41/CD61+ cells from ITP mice by flow cytometry (n = 10). G) The mean fluorescence intensity of IL‐12Rβ2 in CD41/CD61+ cells from ITP mice on day 10 by flow cytometry (n = 10). H) Expression of IL‐35 receptor subunit gp130 in CD41/CD61+ cells from ITP mice by flow cytometry (n = 10). I) The mean fluorescence intensity of gp130 in CD41/CD61+ cells from ITP mice on day 10 by flow cytometry (n = 10). J) The protein levels of IL‐35 receptor subunits IL‐12Rβ2 and gp130 in CD41/CD61+ cells from ITP mice (n = 6). K) The mRNA levels of STAT genes in megakaryocytes (n = 6). L) The mRNA levels of STAT3 and STAT4 were inhibited by si‐STAT3 and si‐STAT4 in CD41/CD61+ cells (n = 6). M) The mRNA levels of IL‐12Rβ2 and gp130 in CD41/CD61+ cells (n = 6). N) The protein levels of IL‐12Rβ2 and gp130 in CD41/CD61+ cells (n = 6). (n = 6). Data information: Data are presented as mean ± SEM. ^*^ *p* < 0.05, ^**^ *p* < 0.01, and ^***^ *p* < 0.001; ns, no significant difference, using ANOVA.

Iguratimod has been shown to suppress the undue immune response. Our group has previously found that iguratimod could increase platelet counts in ITP mice. However, the underlying mechanism was not investigated. Therefore, we went on to evaluate the potential effect of iguratimod on megakaryopoiesis and platelet production. Bone marrow CD34+ cells from ITP patients were differentiated into megakaryocytes with iguratimod or IL‐35. Flow cytometry analysis showed that iguratimod and IL‐35 increased the expression of IL‐12Rβ2 (**Figure** **7**A,B) and gp130 (Figure 7C,D) on megakaryocytes. Iguratimod could better increase the expression of IL‐12Rβ2 than IL‐35 (Figure 7A,B). The expression of gp130 on megakaryocytes was higher in the IL‐35 group than in the iguratimod group (Figure 7C,D). The higher level of IL‐12Rβ2 in the iguratimod group and the higher level of gp130 in the IL‐35 group were confirmed by western blot analysis (Figure 7E). These findings were further confirmed in the ITP mice model. Bone marrow CD34+ cells from WT mice and ITP mice were differentiated into megakaryocytes with and without iguratimod or IL‐35. Flow cytometry analysis indicated that IL‐35 receptors were progressively induced on megakaryocytes from mice, and the expression of IL‐35 receptors was lower in ITP mice than in WT mice (Figure 7F–I). Iguratimod could better increase the expression of IL‐12Rβ2 than IL‐35 (Figure 7F,G). The expression of gp130 was higher in the IL‐35 group than in the iguratimod group (Figure 7H,I). The higher level of IL‐12Rβ2 in the iguratimod group and the higher level of gp130 in the IL‐35 group were confirmed by western blot analysis (Figure 7J).

We further analyzed the mechanisms that control IL‐35 receptor expression in megakaryocytes. It has been previously reported that signal transducer and activator of transcription (STAT) play a role in controlling IL‐35 receptor expression.^[^ ^39^, ^40^, ^41^ ^]^ In our study, the expression of STAT3 and STAT4 were higher in the IL‐35 group and iguratimod group than in the TPO alone group on day 10 (Figure 7K). The expression of STAT3 was higher in the IL‐35 group than in the iguratimod group. The expression of STAT4 was higher in the iguratimod group than in the IL‐35 group. The expression of STAT1 was similar between the different groups. To analyze whether STAT3 or STAT4 was needed for IL‐35 receptor expression during megakaryopoiesis, we knocked down STAT3 and STAT4 using small interfering RNA (siRNA) (Figure 7L). Transfection of CD34+ cells with siRNAs targeting STAT4 resulted in reduced IL‐12Rβ2 mRNA and protein levels in cells stimulated with iguratimod (Figure 7M,N). Transfection of si‐STAT3 decreased gp130 mRNA and protein levels in cells stimulated with IL‐35 (Figure 7M,N). In contrast, si‐STAT3 did not affect IL‐12Rβ2 expression, and si‐STAT4 did not affect gp130 expression. Overall, our data suggests a fundamental role of STAT4 in the induction of IL‐12Rβ2 gene expression and STAT3 in gp130 expression during megakaryocyte differentiation.

### 2.7 Iguratimod Facilitates Platelet Production

**Figure 8:**
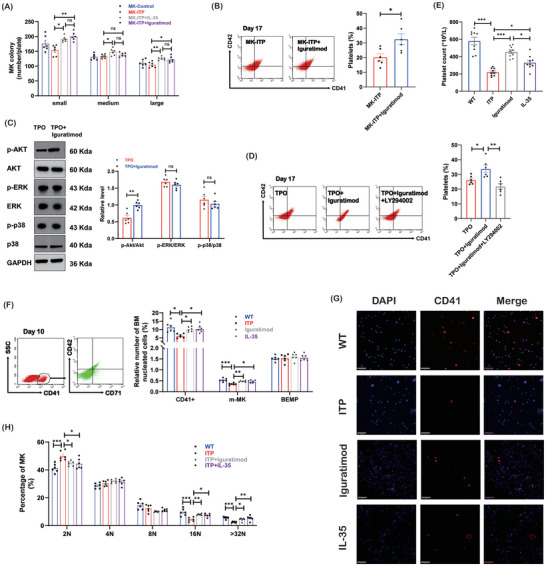
Iguratimod facilitates platelet production. A) The number of megakaryocyte colonies from WT mice, ITP mice, ITP mice treated with IL‐35 or Iguratimod (n = 6). B) Platelet production from megakaryocytes was analyzed by flow cytometry on day 17 (n = 6). C) The protein levels of Akt, ERK, and p38 phosphorylation in megakaryocytes (n = 6). D) Platelet production from megakaryocytes pretreated with Akt phosphorylation inhibitor LY294002 on day 17 (n = 6). E) Platelet counts in WT mice, ITP mice, and ITP mice treated with iguratimod and IL‐35 (n = 9). F) The proportion of bone marrow megakaryocytes in mice was analyzed by flow cytometry (n *=* 6). G) CD41 (red) and DAPI (blue) staining of bone marrow smear of mice (10×; bar: 50 µm). H) The proportion of CD41+ cells of each ploidy in bone marrow from mice (n *=* 6). Data information: Data are presented as mean ± SEM. ^*^ *p* < 0.05, ^**^ *p* < 0.01, and ^***^ *p* < 0.001; ns, no significant difference, using ANOVA (A, D, E, F, H) or Student”s t‐test (B, C).

We went on to investigate the effect of iguratimod on platelet production and related pathways. Iguratimod increased the number of small and large colonies (**Figure** **8**A). There were more platelets released from megakaryocytes in the iguratimod group (Figure 8B). We then evaluated the molecular pathways through which iguratimod regulated platelet production. A higher level of Akt phosphorylation was observed after iguratimod stimulation (Figure 8C). The level of phosphorylation of ERK and p38 was similar between the TPO group and the TPO plus iguratimod group. The Akt phosphorylation inhibitor LY294002 decreased platelet production stimulated by iguratimod (Figure 8D).

The therapeutic efficacy of iguratimod in the murine model was further confirmed. We first evaluated the platelet counts in the ITP murine model after receiving iguratimod treatment. As shown, platelet counts were lower in ITP mice than in WT mice. Iguratimod or IL‐35 increased platelet counts in ITP mice (Figure 8E). To determine whether there was increased megakaryopoiesis in ITP mice treated with iguratimod or IL‐35, the generation of megakaryocytic cells in the bone marrow was assessed. Flow cytometry analysis demonstrated that CD41+ cells were more highly expressed in bone marrow from iguratimod‐ or IL‐35‐treated mice on day 10 (Figure 8F). Furthermore, the percentage of mature megakaryocytes (m‐MK, CD41+/CD42+) was higher in ITP mice receiving iguratimod or IL‐35. However, the percentage of bipotent erythroid‐megakaryocyte progenitors (BEMP, CD41/CD71+) was comparable between the different groups. No difference in the percentage of CD41+ cells, m‐MK, and BMEP was found between the iguratimod group and the IL‐35 group. Immunofluorescence analysis of bone marrow sections showed a decreased megakaryocyte density in ITP mice compared with that in WT mice, which was increased in ITP mice treated with iguratimod or IL‐35 (Figure 8G). Moreover, ploidy was significantly higher in megakaryocytes from ITP mice treated with iguratimod or IL‐35 on day 10 (Figure 8H). The ploidy was similar between the iguratimod group and the IL‐35 group. The results shown above indicated that iguratimod and IL‐35 could promote megakaryopoiesis in the ITP murine model. The platelet counts were higher in the iguratimod group than in the IL‐35 group, which indicated that iguratimod may elevate platelet counts not only by promoting megakaryopoiesis through the IL‐35‐related pathway in vivo.

We then investigated the effects of ITP‐MSCs on T‐cell differentiation and proliferation in the presence of iguratimod. There was no difference in T‐cell subsets between the groups with and without iguratimod (Figure S2A, Supporting Information). The expression of FAM107A was comparable between the two groups (Figure S2B, Supporting Information). Thus, we postulated that iguratimod did not decrease the enhanced apoptosis of ITP‐MSCs and did not restore their immunosuppressive capacity. To verify the hypothesis, we cocultured ITP‐MSCs with iguratimod. CCK8 assays showed that iguratimod did not affect the proliferation of ITP‐MSCs (Figure S2C, Supporting Information). The rate of apoptosis was similar between the groups with and without iguratimod (Figure S2D, Supporting Information). At the protein level, the Bcl‐2/Bax ratio and the levels of caspase‐9 and caspase‐3 were not affected by iguratimod (Figure S2E, Supporting Information). We then determined the concentration of cytokines in coculture supernatants. Iguratimod did not reverse the decreased TGF‐β and increased MCP1 and IL‐6 in the coculture system (Figure S2F, Supporting Information). The level of IL‐35 in the coculture supernatant was also comparable between the groups with and without iguratimod (Figure S2F, Supporting Information).

### 2.8 Clinical Outcomes of Iguratimod in Corticosteroid‐Resistant ITP Patients

A retrospective study was conducted to evaluate the efficacy and safety of iguratimod in corticosteroid‐resistant ITP patients. A total of 68 adult patients with corticosteroid‐resistant primary ITP were finally included in our study. The baseline characteristics of the patients are shown in **Table** **1**. There were 33/68 (49%) male patients. The median age was 38.6 years, and the median duration of ITP was 35 months. The baseline platelet count was 15.5 × 10^9^ L^−1^. Bleeding was observed in 37/68 (54%) patients. All enrolled subjects had concomitant treatments, including corticosteroids in 58/68 (85%) patients, intravenous immunoglobulin (IVIG) in 16/68 (24%) patients, and thrombopoietin receptor agonists (TPO‐RAs) in 33/68 (49%) patients.

**Table 1:** Baseline characteristics of corticosteroid‐resistant immune thrombocytopenia patients.

	ITP patients [n = 68]
Age, median (IQR)	38.6 (21.0‐54.0)
Gender (male), n (%)	33 (49%)
Duration from diagnosis (months), median (IQR)	35 (20‐76)
Baseline platelet counts (10^9^ L^−1^)	15.5 (8.0‐24.5)
WHO bleeding score	
0	31 (46%)
1	21 (31%)
2	10 (15%)
3	6 (9%)
4	0
Concomitant treatments, n (%)	68 (100%)
Corticosteroids	58 (85%)
IVIG	16 (24%)
TPO‐RAs	33 (49%)
Cyclosporin	2 (3%)
Rituximab	2 (3%)
Other	13 (19%)

Abbreviation: IQR, Interquartile range; IVIG, intravenous immunoglobulin; TPO‐RAs, thrombopoietin receptor agonists.

The outcomes at 6 months are summarized in **Table** **2**. Initial response was achieved in 42/68 (62%) patients, and complete response was achieved in 25/68 (37%) patients. In patients who achieved an initial response, the median time to respond was 28 days, and the peak platelet count was 170 × 10^9^ L^−1^. During the 6‐month follow‐up, 7/42 (17%) patients relapsed. A sustained response was seen in 35/68 (51%) patients. Bleeding symptoms were reduced significantly and were seen in 14/68 (21%) patients at 6 months. All subjects had concomitant treatments at enrollment, and 25/68 (37%) patients reduced or discontinued concomitant medication. Rescue therapy was seen in 19/68 (28%) patients, and the most common rescue treatments were corticosteroids (5/68, 7%) and platelet transfusion (5/68, 7%). The median platelet counts in each week are shown in Figure S3 (Supporting Information).

**Table 2:** Clinical outcomes at 6 months.

	ITP patients [n = 68]
Efficacy	
Initial response, n (%)	42 (62%)
Complete response, n (%)	25 (37%)
Patients with initial response	
Time to response (days), median (IQR)	28 (12‐50)
Peak platelet counts (10^9^ L^−1^), median (IQR)	170 (102.6‐208.0)
Sustained response, n (%)	35 (51%)
Bleeding symptoms, n (%)	14 (21%)
Rescue therapy, n (%)	19 (28%)
Corticosteroids	5 (7%)
IVIG	3 (4%)
Splenectomy	0
Rituximab	4 (6%)
Platelet transfusion	5 (7%)
TPO‐RAs	2 (3%)

Abbreviation: ITP, immune thrombocytopenia; IQR, Interquartile range; IVIG, intravenous immunoglobulin; TPO‐RAs, thrombopoietin receptor agonists.

No grade 4 or worse adverse events occurred in our patients. The most common adverse events of iguratimod were elevated ALT (7/68, 10%), gastrointestinal disorders (6/68, 9%), and nausea (5/68, 7%) (**Table** **3**). Most adverse events were mild (grade 1–2). Only one patient discontinued iguratimod because of adverse events (elevated ALT of grade 3). No other patients reduced or discontinued iguratimod due to side effects.

**Table 3:** Adverse events of iguratimod.

	ITP patients [n = 68]	
	Grade 1–2	Grade 3
Elevated ALT, n (%)	7 (10%)	1 (1.5%)
Elevated AST, n (%)	4 (6%)	0
Elevated γ‐GGT, n (%)	2 (3%)	0
Decreased leukocytes, n (%)	2 (3%)	0
Decreased hemoglobin, n (%)	1 (1%)	0
Diarrhea, n (%)	2 (3%)	0
Headache, n (%)	3 (4%)	0
Lethargy, n (%)	1 (1%)	0
Gastrointestinal disorders, n (%)	6 (9%)	0
Nausea, n (%)	5 (7%)	0
Rash, n (%)	2 (3%)	0

Abbreviation: ITP, immune thrombocytopenia.

## 3 Discussion

We have shown that the level of IL‐35 in bone marrow was reduced in ITP patients compared with that in healthy controls. The IL‐35 receptor was expressed on megakaryocytes during in vitro megakaryopoiesis, and the expression of the IL‐35 receptor was downregulated in megakaryocytes from ITP. IL‐35 induced the expression of the IL‐35 receptor and increased the number of megakaryocyte colonies. Our findings indicated that IL‐35 played a significant role in the pathogenesis of ITP.

Apart from excessive destruction of platelets, another pathophysiology of ITP is the impaired megakaryopoiesis and platelet production caused by autoantibodies and cytotoxic T cells.^[^ ^42^ ^]^ Intrinsic defects in megakaryocytes and extrinsic inflammatory environments have been implicated in altered megakaryopoiesis in ITP. In this study, we focused on the effect of IL‐35 on megakaryopoiesis.

IL‐35 functions through IL‐35 receptors, which consist of the IL‐12Rβ2 and gp130 subunits. To examine the function of IL‐35 in megakaryopoiesis, we first assessed the expression of IL‐35 receptors on megakaryocytes. We found that the expression of the IL‐35 receptor was progressively induced on megakaryocytes. In contrast, the level of the IL‐35 receptor was significantly decreased in megakaryocytes from ITP patients, which implied that IL‐35 might play an important role in impaired megakaryopoiesis. Then, we further induced the differentiation of megakaryocytes using IL‐35. We showed that culture with IL‐35 promoted the differentiation of megakaryocytes through the Akt pathway in vitro. The primary type of cells that secrete IL‐35 is iTr35, and other immune cells, including Tregs, Bregs, and dendritic cells, also produce IL‐35.^[^ ^1^, ^4^, ^6^, ^7^, ^8^, ^9^, ^10^, ^11^ ^]^ We found that the number of iTr35 cells decreased in ITP bone marrow, which might explain the reduced level of IL‐35 in ITP bone marrow. Megakaryopoiesis involves numerous specialized intracellular signaling pathways, which have essential roles in cellular proliferation and differentiation.^[^ ^43^ ^]^ Our data suggest that IL‐35 can promote thrombopoiesis, which is modulated via Akt signaling.

MSCs are multipotent stem cells with immunomodulatory properties.^[^ ^36^ ^]^ Given the immunomodulatory effect of MSCs, we cocultured CD4+ T cells with bone marrow MSCs from healthy donors and ITP patients. Our results showed that ITP‐MSCs exhibited an impaired capability of inducing iTr35. The level of TGF‐β, one of the most commonly studied immunosuppressive cytokines, was lower in coculture supernatants in the ITP‐MSCs group. Our data indicated that TGF‐β may play a pivotal role in the induction of iTr35, which warrants further investigation. To search for potential targets, we characterized the expression levels of lncRNAs in MSCs from ITP patients and healthy controls using RNA sequencing. It has been demonstrated that the dysregulation of gene regulation participates in the pathogenesis of ITP.^[^ ^44^ ^]^ Accumulating evidence indicates that lncRNAs might monitor the progression of ITP and serve as intervention targets. Previous studies have investigated the role of lncRNAs in regulating the functions and differentiation of lymphocytes and hence their role in the pathology of ITP.^[^ ^45^, ^46^ ^]^ LncRNAs act through targeted genes to mediate their actions and influence the development of ITP.^[^ ^47^ ^]^ For example, targeting STAT3 by lncDC affected the differentiation of dendritic cells, which then induced the activation of the immune response.^[^ ^48^, ^49^ ^]^ However, the exact molecular mechanisms of many lncRNAs in ITP remain unclear. Therefore, we investigated the aberrantly expressed lncRNAs and the associated TF‐mRNA network in ITP‐MSCs. We identified that FAM107A was overexpressed in ITP‐MSCs. FAM107A is a candidate tumor suppressor gene located on chromosome 3p21.1 that encodes a protein of 144 amino acids.^[^ ^50^ ^]^ FAM107A has dual functions in tumor biological behaviors, including inducing apoptosis in early tumors and promoting invasion.^[^ ^38^, ^51^ ^]^ In terms of FAM107A mechanisms, we demonstrated that FAM107A plays an essential role in regulating the immunomodulatory function of ITP‐MSCs, at least in part, through promoting apoptosis. Further studies are needed to confirm our findings and assess the importance of FAM107A in ITP.

The immune modulation effects of iguratimod have made it a widely used drug for rheumatic diseases. Clinical trials have confirmed that the concomitant use of iguratimod and methotrexate is safe and effective in RA patients.^[^ ^24^ ^]^ Iguratimod suppressed the proliferation of Th17 cells and secretion of inflammatory cytokines and upregulated Tregs in RA patients.^[^ ^34^ ^]^ Iguratimod ameliorated proteinuria in murine nephritis of SLE, modulated the ratio of Th17/Treg, and increased the serum levels of TGF‐β and IL‐10.^[^ ^52^ ^]^ Iguratimod alleviated the clinical symptoms of patients with pSS, increased platelet counts, and ameliorated the secretory function of salivary and lacrimal glands.^[^ ^53^ ^]^ The proportions of peripheral plasma cells decreased after treatment with iguratimod.^[^ ^54^ ^]^ T and B cells play critical roles in the underlying pathogenesis of ITP, implying that iguratimod may have the potential for the treatment of ITP. Retrospective results in our study indicated that the initial response rate of iguratimod was 62% and the complete response rate was 37% in corticosteroid‐resistant ITP patients. In the 6‐month follow‐up, 10% of patients relapsed, and rescue therapies were used in 28% of patients. In the safety assessment, previous reports did not observe severe side effects of iguratimod.^[^ ^54^ ^]^ Gastrointestinal discomfort has been reported to be the most common adverse event.^[^ ^55^ ^]^ In our study, only one patient had elevated ALT of grade 3, and there were no grade 4 or worse adverse events. The most common adverse events in our patients were elevated ALT, gastrointestinal disorders, and nausea, which did not lead to the discontinuation of this drug. Our results further demonstrated the safety of iguratimod.

Several limitations should be acknowledged in our study. First, the results of bioinformatics analysis of the interaction between lncRNAs, TFs, and mRNAs should be further validated. Second, we did not explore the underlying mechanism of ITP‐MSCs in inducing T‐cell differentiation. More in‐depth investigations are warranted to determine the potential molecular pathways. Third, the sample size of the clinical study of iguratimod was small, and our current study had a short follow‐up period. A prospective study in a larger ITP population with a longer follow‐up duration would offer a stronger appreciation of the clinical outcomes of iguratimod in ITP patients. In addition, single‐cell RNA sequencing analyses of bone marrow MSCs still lack functional verification.

In conclusion, this study demonstrates that IL‐35 receptors are progressively expressed on megakaryocytes and that IL‐35 promotes megakaryopoiesis through the Akt pathway. ITP‐MSCs exhibited an impaired capability of inducing iTr35 due to enhanced apoptosis, which might contribute to the reduced level of bone marrow IL‐35 in ITP patients. Iguratimod is capable of inducing the expression of IL‐35 receptors on megakaryocytes and promoting megakaryopoiesis. Iguratimod improves the response rate and reduces bleeding symptoms in ITP patients.

## 4 Experimental Section

### Patients and Study Design

Bone marrow samples from adult patients with treatment‐naive primary ITP at Peking University People's Hospital were included in this study from June 2021 to July 2022.^[^ ^56^, ^57^ ^]^ Gender‐ and age‐matched bone marrow donors were included as healthy controls. The baseline characteristics of ITP patients and controls in our study are described in Table S1 (Supporting Information). All enrolled subjects provided written informed consent in accordance with the Declaration of Helsinki. The study protocol was assessed and approved by the Ethics Committee of Peking University People's Hospital (2020PHB304).

In the retrospective study of iguratimod, corticosteroid‐resistant ITP patients who had been treated with iguratimod from February 2020 to December 2021 were evaluated. Inclusion criteria were: 1) primary ITP diagnosis; 2) age ≥ 18 years; 3) previous iguratimod treatment; 4) concomitant regimens for ITP were allowed, provided the doses had remained stable for at least 4 weeks before receiving iguratimod; 5) patients had at least 6 months of follow up after beginning iguratimod. Patients with secondary thrombocytopenia were excluded. Patients usually receive 25 mg of oral iguratimod twice daily. Patients were retrospectively reviewed to collect demographic and clinical data, including platelet counts, bleeding symptoms, and adverse events during iguratimod treatment. The definitions of initial response, complete response, relapse, duration of response, and time to response are detailed in Table S2 (Supporting Information). Any increase in concomitant therapy for primary ITP to be rescue treatment was considered, and patients who received rescue treatment were considered to be non‐responders.

### Construction of the ITP Mice Model

The active ITP mice model according to the previous studies were constructed.^[^ ^58^, ^59^ ^]^ Briefly, 10^8^ platelets obtained from wild‐type (WT) C57BL/6 mice (6–8 weeks) were transfused into C57BL/6 CD61 KO mice via the caudal vein weekly for 6 consecutive weeks. On the day of splenocyte transfer, SCID mice were subjected to 200 cGy total body irradiation. Within 3 h of irradiation, SCID mice were injected intraperitoneally with 2 × 10^4^ splenocytes from immunized CD61 KO mice. Profound thrombocytopenia and bleeding manifestations (gastrointestinal and subcutaneous) were observed in the constructed murine model for 7 days after the transfusion of splenocytes, indicating a successful active ITP model. Mice were intraperitoneally injected with 200 ng d^−1^ IL‐35 (Chimerigen, Liestal, Switzerland) and were gavaged with 30 mg kg^−1^ day^−1^ iguratimod (Selleck, TX, USA) for 10 days, respectively. The animal experiments adhered to the Institutional Guidelines for the Care and Use of Laboratory Animals and were approved by the Ethics Committee of Peking University People's Hospital (2021PHE112).

### Isolation and Culture of Bone Marrow MSCs

MSCs were isolated by Ficoll gradient (specific gravity 1.077 g mL^−1^; Sigma, St. Louis, MO, USA) following the previous publications.^[^ ^37^, ^58^ ^]^ The isolated cells were cultured at 10^7^ cells ml^−1^ in Dulbecco's modified Eagle's medium (Gibco, Carlsbad, CA, USA) supplemented with 10% fetal bovine serum (Gibco). The culture medium was placed in a 5% CO_2_ chamber at 37 °C and was exchanged every 3 to 4 days. MSCs were passaged after reaching 80% confluence. After reaching the third passage, the MSCs were plated into 96‐well culture dishes in culture medium. The expression of surface‐specific antigens was analyzed using flow cytometry (Beckman Coulter, CA, USA) to confirm the phenotype of MSCs with the following antibodies: CD34 (34‐581‐18), CD45 (14‐0451‐82), CD90 (17‐0909‐42), CD105 (12‐1057‐42), and HLA‐DR (11‐9956‐42) (all from Thermo Scientific, MA, USA). The capacity of the MSCs to differentiate along osteogenic and adipogenic lineages was assessed with commercial kits according to the manufacturer's instructions.

### Coculture Experiments and Analysis of Tregs

MSCs were seeded at 5 × 10^4^ cells L^−1^ in 24‐well plates for 24 h before the coculture in Roswell Park Memorial Institute (RPMI) 1640 medium (Sigma) according to the previous report.^[^ ^37^ ^]^ CD4+ T cells isolated from the peripheral blood mononuclear cells of normal controls were purified using positive selection with a CD4+ T‐cell isolation kit (MicroBeads; Miltenyi Biotec, Bergisch Gladbach, Germany). Then, CD4+ T cells were seeded above the MSCs layer at a ratio of 10:1 T cells/MSCs. To investigate the effect of iguratimod, iguratimod (100 µm) was added to the cultures at day 0. After 5 days of coculture, nonadherent cells were harvested to evaluate the proportion of Tregs by flow cytometry using PE‐Cy7‐conjugated anti‐CD4, PE‐conjugated anti‐CD25, and antigen‐presenting cell‐conjugated anti‐Foxp3 antibodies (all from BD Biosciences, CA, USA). The supernatants were collected to conduct cytokine release analysis by ELISA.

### Isolation and Culture of CD34+ Cells

CD34+ cells were isolated by Ficoll gradient following our previous publications.^[^ ^37^, ^58^ ^]^ Mononuclear cells were isolated using Ficoll Hypaque density centrifugation (Centrifuge 5415D, Eppendorf, Westbury, NY, USA). The isolated cells were plated at 10^7^ cells mL^−1^. After 90 min, the non‐attached cells were collected and incubated with microbead‐conjugated anti‐CD34 monoclonal antibody for 20 min at 4 °C, and then processed using a magnetic separation column (Miltenyi Biotec) to collect CD34+ cells. CD34+ cell preparations were analyzed by flow cytometry using the single platform CD34 assay kit (Stem‐Kit, Beckman Coulter, CA, USA) to measure the direct counts of CD34+ cells.

### In Vitro Differentiation of Megakaryocytes

For megakaryocyte differentiation, bone marrow CD34+ cells were grown in serum‐free medium (IMDM) at a density of 5 × 10^4^ cells ml^−1^ and differentiated into megakaryocytes with thrombopoietin (20 ng ml^−1^, 78 210, StemCell, Vancouver, BC, Canada). Cells were cultured at 37 °C in a 5% CO_2_ humidified chamber and collected at different time points. To estimate the effects of iguratimod on the differentiation of megakaryocytes, iguratimod (100 µm) was added to the culture medium.^[^ ^29^ ^]^ To estimate the effects of IL‐35, IL‐35 (10 ng mL^−1^) was supplemented into the culture medium.^[^ ^60^ ^]^ Cells were labeled with an anti‐CD41/CD61 antibody and an anti‐IL‐12Rβ2 antibody or anti‐gp130 antibody (all from BD Biosciences). The washed cells were suspended in PBS containing 7‐AAD. The percentage of positive staining cells was determined using flow cytometry, and the median fluorescence intensity (MFI) was based on the gated positive cell population of each megakaryocyte marker. To determine the ploidy status, cells were stained with both Hoechst 33 342 (Invitrogen, Carlsbad, CA, USA) and anti‐CD41 antibody (ab134131, abcam), and flow cytometry was performed.

### Isolation of Culture‐Derived Platelets

To determine the level of culture‐derived platelets, CD34+ cell‐derived megakaryocytes were centrifuged at 200 g for 5 min, and the supernatant was centrifuged a second time at 1550 g for 10 min. The supernatant was discarded and the platelet‐containing pellets were labeled with APC‐CD41 and PE‐CD42 antibodies in PBS (all from BD Biosciences). After incubation for 30 min in the dark at room temperature, cells were analyzed using a flow cytometer.

### Platelet Aggregation Experiments

The centrifuged pellets containing the platelets were adjusted to 30 × 10^4^ platelets µL^−1^. For platelet aggregation studies, platelet‐rich samples were preincubated with 5 µm of adenosine diphosphate (ADP, Sigma), 15 mm arachidonic acid (AA, Sigma), and 2 mg mL^−1^ of collagen (Sigma). The percent aggregation of platelets was determined by an aggregometer (Helena Laboratories, TX, USA).

### Immunofluorescence Assay

Megakaryocytes were washed once with PBS and fixed in 4% paraformaldehyde for 15 min and then blocked with 5% bovine serum albumin at room temperature for 2 h. The cells were incubated with primary antibodies against IL‐12Rβ2 (MAB19591‐SP, R&D Systems, MN, USA), gp130 (MA5‐38467, Thermo, MA, USA), β‐tubulin (ab6046, abcam), CD41 (ab134131, abcam) overnight at 4 °C, followed by fluorescein isothiocyanate‐conjugated goat anti‐rabbit IgG antibody (ab6717, abcam) and DAPI for nuclear counterstaining (Vectashield) at room temperature for 1 h. Samples were mounted in FluorSave Reagent (Calbiochem, San Diego, CA, USA). Pictures were obtained using an IX71 Olympus Fluorescence Microscope equipped with a digital camera or a Nikon ECLIPSE Ti Confocal Laser‐Scanning Microscope.

### Colony‐Forming Units Assay

Megakaryocytes were cultured on a semisolid serum‐free medium (04961, StemCell) at 37 °C and 5% CO_2_. The colonies were quantified after dehydration, fixation, and staining according to the manufacturer's instructions. The colonies were counted under a microscope. The cluster of three or more megakaryocytes was scored as a colony. The colonies with 3–20 cells per colony were rated as small colonies. The colonies with 21–50 cells per colony were rated as medium‐sized colonies. The colonies with >50 cells per colony were rated as large colonies.

### Enzyme‐Linked Immunosorbent Assay

IL‐35 levels were assessed in the plasma, bone marrow supernatants, and culture supernatants via commercial ELISA kits (abx252675, Abbexa, Cambridge, UK) according to the manufacturer's instructions. The commercial ELISA kits were used to measure IL‐2 (ELH‐IL2‐2, Raybiotech, GA, USA), IL‐6 (ELH‐IL6‐5, Raybiotech), IL‐10 (ELH‐IL10‐1, Raybiotech), IL‐12 (DG10297H, Dogesce, Beijing, China), IL‐17A (DG12513H, Dogesce), IL‐21 (ELH‐IL21‐1, Raybiotech), IDO (NBP2‐62765, Novus, MO, USA), PGE2 (ADI‐900‐001, Enzo, NY, USA), MCP1 (ELH‐MCP1‐2, Raybiotech), and TGF‐β (DG10113H, Dogesce) in the culture supernatants.

### Western Blot

Proteins from cells were prepared using a lysing buffer according to our previous protocols.^[^ ^37^, ^58^ ^]^ The concentration was determined with a BCA Protein Assay kit (Solarbio, Beijing, China). Proteins were fractionated by SDS‐PAGE gels and transferred to the PVDF membrane (Millipore, Boston, MA, USA). After being blocked with 5% fat‐free milk for 2 h at room temperature, the proteins were incubated with primary antibodies against IL‐12Rβ2 (MAB19591‐SP, R&D Systems), gp130 (MA5‐38467, Thermo), β‐tubulin (ab6046, abcam), CD41 (ab134131, abcam), Akt (4685S, CST, MA, USA), p‐Akt (13 038, CST), ERK (4695S, CST), p‐ERK (ab214036, abcam), p38 (8690, CST), p‐p38 (9216S, CST) overnight at 4 °C, followed by the secondary antibodies at room temperature for 1 h. Proteins were visualized using a chemiluminescent peroxidase substrate. The relative expression of the proteins was normalized to GAPDH. A quantitative analysis was performed by scanning the blots and calculating the relative intensities in relation to the corresponding GAPDH signal using Quantity One (Bio‐Rad, Laboratories, Hercules, CA, USA).

### Real‐Time PCR

Total RNA from cultured cells was prepared using Trizol Reagent (Invitrogen) and processed using the RNeasy kit (Qiagen, Valencia, CA, USA) according to the manufacturer's instructions. Total RNA from each sample was reverse transcribed into complementary DNA (cDNA) using Qiagen Omniscript according to the manufacturer's protocol. Real‐time PCR was performed in 20 µL reaction system, which included 1 µL of cDNA, 10 µL of Hieff qPCR SYBR Green Master Mix (Applied Biosystems, Foster City, CA, USA), and 250 nm of each primer, and conducted in the MA‐6000 RT‐PCR System (Molarray, Suzhou, China). Reactions were performed in triplicate, and the relative expression levels were normalized to GAPDH using the 2^−ΔΔ^ *^C^* ^t^ method.

### RNA Sequencing Analysis

Illumina HiSeq system (San Diego, CA, USA) was employed for transcriptome profiling to detect the expression profiles of mRNA and lncRNA in bone marrow MSCs isolated from two different healthy donors and four different ITP patients. Base calling was conducted using real‐time analysis. Fastq files were mapped to the human genome by TopHat (version 2.1.1, Baltimore, MD, USA). Mapped reads were assembled with Cufflinks (version 2.2.1, WA, USA), and then merged with the Cuffmerge program. The mRNA and lncRNA levels were calculated with the Cuffdiff program, with three replicates in each group. Differentially expressed genes between groups were defined as those whose expression was statistically different (p <0.05) by at least twofold change, and were ranked from most upregulated to most downregulated. To better analyze the functions of differentially expressed lncRNA, the Gene Ontology (GO) and Kyoto Encyclopedia of Genes and Genomes (KEGG) pathway analyses were conducted to classify the enriched functional pathways. The lncRNA‐transcription factor (TF)‐mRNA network was built by overlapping target mRNAs of the lncRNA with the target genes of predicted TFs. The lncRNA‐TF‐mRNA regulatory network with Cytoscape (version 3.7.2) software was constructed and visualized.

### Statistical Analysis

Student's t‐test, one‐way analysis of variance (ANOVA), two‐way ANOVA, Mann‐Whitney test, and χ2 tests were employed as appropriate. Data were presented as mean ± standard error of the mean (SEM), or mean ± standard deviation (SD), or median (interquartile range, IQR) as appropriate. A *p* <0.05 was considered statistically significant (* *p* < 0.05, ^**^ *p* < 0.01, and ^***^ *p* < 0.001; ns, no significant difference). Statistical analyses were conducted using SPSS software 22.0 (IBM, Armonk, NY, USA) and GraphPad Prism 7 (GraphPad Software, USA).

### Ethics Approval

The study was approved by the Ethics Committee of Peking University People's Hospital (2020PHB304), and written informed consent was obtained according to the Declaration of Helsinki. Animal experiments were undertaken in accordance with the Institutional Guidelines for the Care and Use of Laboratory Animals, and approved by the Ethics Committee of Peking University People's Hospital (2021PHE112).

## Conflict of Interest

The authors declare no conflict of interest.

## Supporting information

Supporting Information

## Data Availability

The data that support the findings of this study are available from the corresponding author upon reasonable request.
