# Supplementary material for: Decreased Expression of IL‐35 and Its Receptor Contributes to Impaired Megakaryopoiesis in the Pathogenesis of Immune Thrombocytopenia
Source: Adv Sci (Weinh). 2024 Jan 15;11(12):2305798. doi: 10.1002/advs.202305798 (PMC10966519; doi:10.1002/advs.202305798)
Supplement: Supplementary file 1 — Supporting Information [file ADVS-11-2305798-s001.pdf]

ADVANCED SCIENCE
Open Access

Supporting Information

for *Adv. Sci.*, DOI 10.1002/advs.202305798

Decreased Expression of IL-35 and Its Receptor Contributes to Impaired Megakaryopoiesis in the Pathogenesis of Immune Thrombocytopenia

*Xuan Cai, Ruo-Yun Gui, Jin Wu, Chen-Cong Wang, Xiao-Lu Zhu, Hai-Xia Fu and Xiao-Hui Zhang**

Supporting Information

Decreased Expression of IL-35 and Its Receptor Contributes to Impaired Megakaryopoiesis in the Pathogenesis of Immune Thrombocytopenia

*Xuan Cai,[1,2,3,4†] Ruo-yun Gui,[1,2,3,4†] Jin Wu,[1,2,3,4] Chen-Cong Wang,[1,2,3,4] Xiao-Lu Zhu,[1,2,3,4] Hai-Xia Fu,[1,2,3,4] Xiao-Hui Zhang[1,2,3,4]**

Supplementary Figures

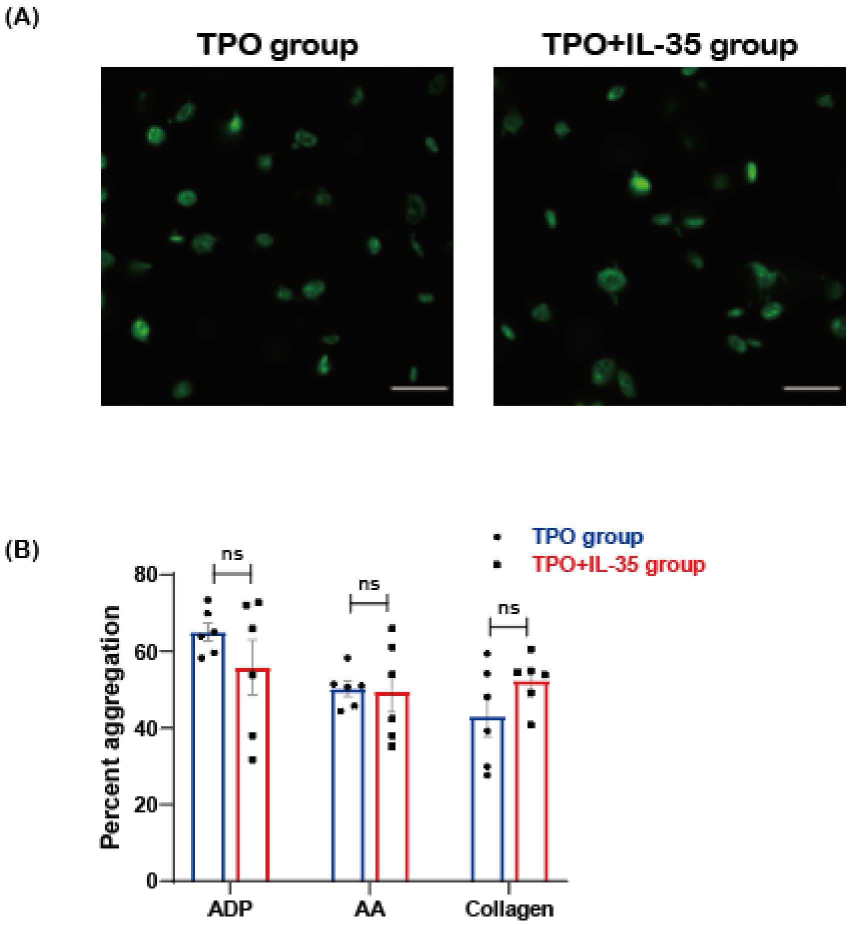

Figure S1. The size and function of platelets. (A) Immunofluorescence image of platelets stained with antibodies to β-tubulin in the TPO group and the TPO+IL-35 group (200×; bar: 20 μm). (B) The percent aggregation of platelets in response to ADP, AA, and collagen in the TPO group and the TPO+IL-35 group. Data information: Data are presented as mean ±SEM. ns, no significant difference, using Student's t-test.

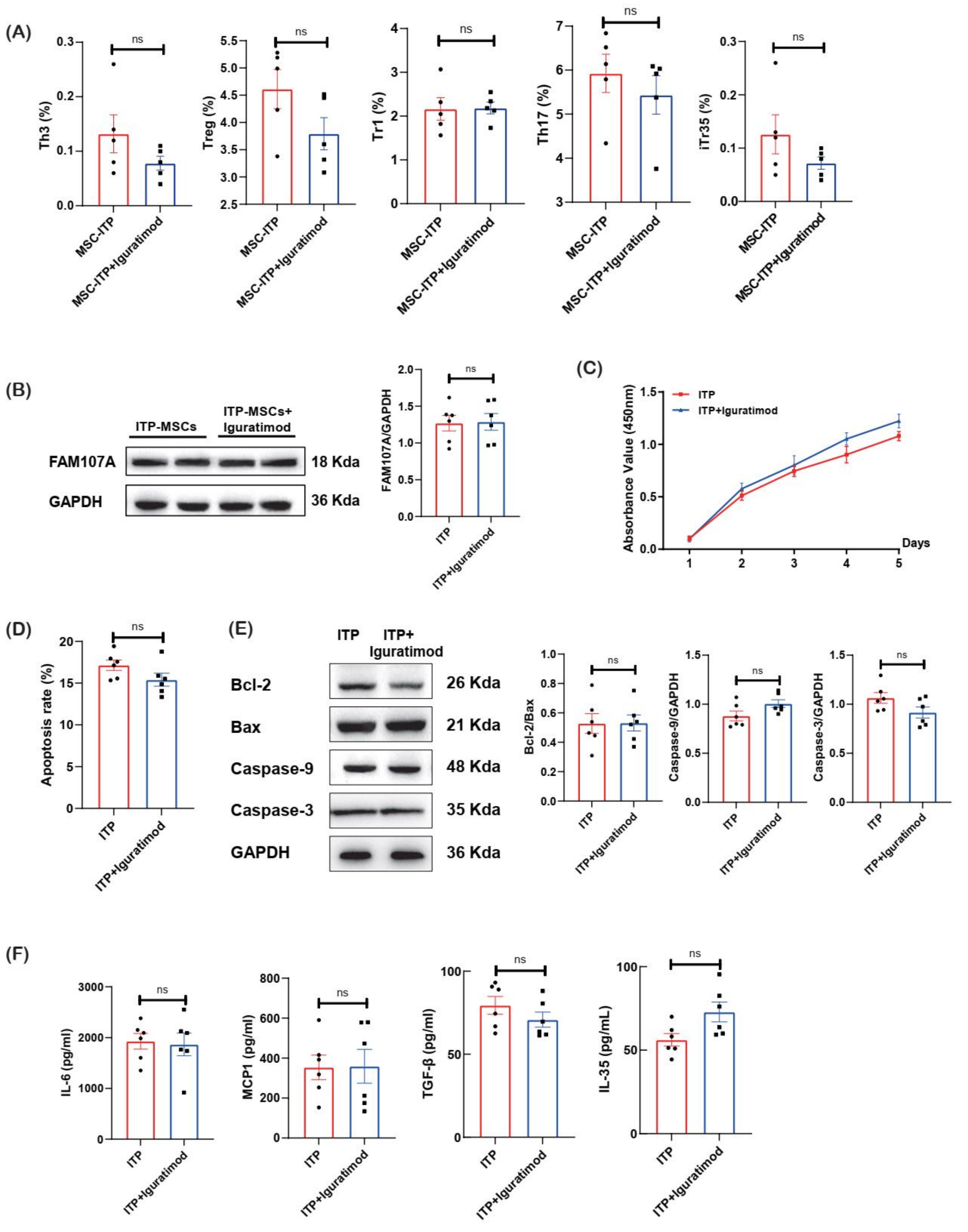

Figure S2. Iguratimod does not reverse the impaired immunomodulatory effects of ITP-MSCs. (A) Flow cytometry analysis of T-cell subsets (n=5). (B) The protein levels of FAM107A in MSCs (n=6). (C) Growth curves of MSCs (n=6). (D) The apoptotic cell rate of ITP-MSCs determined by flow cytometry (n=6). (E) The protein levels of apoptosis markers in MSCs (n=6). (F) The concentration of cytokines in the coculture supernatant of T-cells and MSCs (n=6). Data information: Data are presented as mean ± SEM. * P <0.05, ** P <0.01, and *** P <0.001; ns, no significant difference, using Student's t-test (A, B, D, E, F) or ANOVA (C).

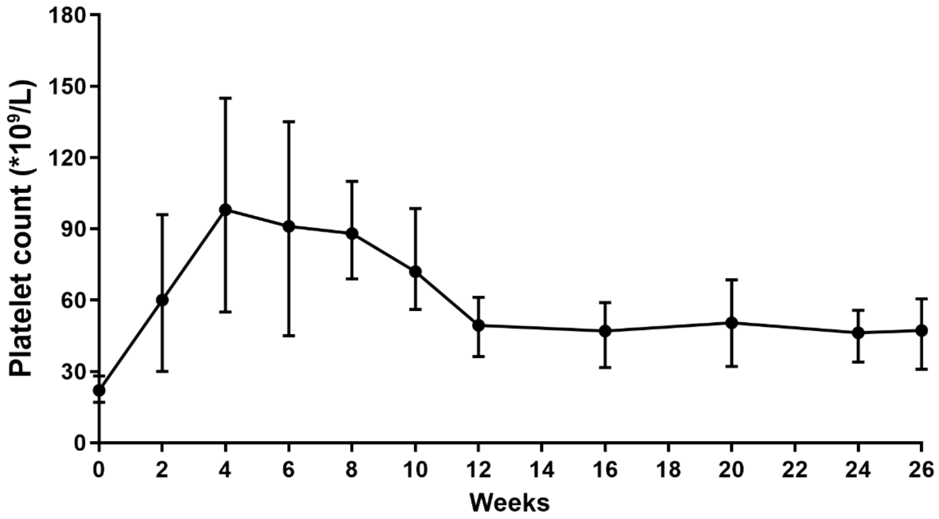

Figure S3. Median platelet counts by study week in ITP patients who received iguratimod. Error bars represent the IQR.

Supplementary tables

Table S1. Baseline characteristics of the enrolled subjects.

	ITP patients	Controls	*p*
Gender (male)	24 (48%)	16 (48%)	0.935
Age	46.5 (19-62)	46.7 (26-58)	0.975
Platelet ($\times 10^9$ L^{-1})	38 (8-74)	195 (142-313)	< 0.001
WBC ($\times 10^9$ L^{-1})	6.1 (3.9-8.6)	6.7 (4.6-9.0)	0.764
Hemoglobulin (g L^{-1})	138 (118-165)	144 (121-177)	0.551
Bleeding	22 (44%)	-	-

Abbreviation: ITP, immune thrombocytopenia; WBC, white blood cells.

Table S2. Clinical definitions.

	Definitions
Corticosteroid-resistant ITP	Patients who did not achieve a sustained response to treatment with full-dose corticosteroids for a minimum duration of 4 weeks or had relapsed during steroid-tapering or after its discontinuation.
Sustained response	The maintenance of platelet count ≥ 30×10^9 L^{-1}, at least 2-fold increase of the baseline count, the absence of bleeding, and no need for rescue medication at the 6-month follow-up.
Response	The maintenance of platelet count ≥ 30×10^9 L^{-1}, at least 2-fold increase of the baseline count, the absence of bleeding, and no need for rescue medication.
Complete response	The maintenance of platelet count ≥ 100×10^9 L^{-1}, at least 2-fold increase of the baseline count, the absence of bleeding, and no need for rescue medication.
Time to response	The time period from starting treatments to achieving response.
Duration of response	The time period from the achievement of response to the loss of response.

Abbreviation: ITP, immune thrombocytopenia.
